# Draft Genome Sequences of Basidiomycetous Epiphytic Phylloplane Yeast Type Strains *Dioszegia crocea* JCM 2961 and *Dioszegia aurantiaca* JCM 2956

**DOI:** 10.1128/MRA.01727-18

**Published:** 2019-06-27

**Authors:** Masako Takashima, Ri-ichiroh Manabe, Moriya Ohkuma

**Affiliations:** a Japan Collection of Microorganisms, RIKEN BioResource Research Center, Tsukuba, Ibaraki, Japan; b Division of Genomic Technologies, RIKEN Center for Life Science Technologies, Yokohama, Kanagawa, Japan; University of Maryland School of Medicine

## Abstract

We report the draft genome sequences of type strains for Dioszegia crocea and its closely related species Dioszegia aurantiaca, which should improve our understanding of the epiphytic phylloplane yeasts. These data will also have implications for the plant microbiome, since Dioszegia is considered a microbial “hub” taxon.

## ANNOUNCEMENT

Members of the genus Dioszegia (Tremellales, Agaricomycotina, Basidiomycota) are known as epiphytic phylloplane yeasts characterized by orange-yellow or orange to deep-orange colonies and the production of ballistoconidia (1). Most of the Dioszegia species have been isolated from plant leaves, although more recently, some have also been found in soil and in water. A study by Renker et al. (2), in which a sequence identical to the internal transcribed spacer (ITS) region of Dioszegia crocea was observed in DNA isolated from the roots and spores of arbuscular mycorrhizal fungi, suggested the possibility of a close relationship between plants and *Dioszegia* species.

To contribute to the understanding of epiphytic phylloplane yeasts, we determined the draft genome sequence for type strains of D. crocea (JCM 2961, isolated from a strawberry and originally described as Bullera crocea) (3) and its phylogenetically closely related species Dioszegia aurantiaca (JCM 2956, isolated from overwintered nettle stems of Urtica spp. and originally described as Bullera aurantiaca) (4); both are now classified as members of the genus *Dioszegia* (5). Strains obtained from the Japan Collection of Microorganisms (JCM), RIKEN BioResource Research Center, were cultivated on YM agar (glucose 10.0 g/liter, peptone 5.0 g/liter, yeast extract 3.0 g/liter, malt extract 3.0 g/liter, agar 20.0 g/liter; BD Difco) plates at 25°C, and the cells were harvested. The genomic DNA was prepared from freeze-dried cell pellets, according to the method of Raeder and Broda (6), and purified using a Genomic-tip 100/G kit (Qiagen, Tokyo), following the manufacturer’s instructions. Paired-end and mate pair libraries with approximate insert sizes of 240 bp and 3 kbp, respectively, were prepared from DNA using a TruSeq DNA PCR-free library preparation kit and a Nextera mate pair sample preparation kit (Illumina, CA), respectively, with some modifications (7). Genome sequencing was accomplished on a HiSeq 2500 platform (Illumina). The reads from mate pair libraries were processed with NextClip v0.8 (8) to trim adapter sequences. ALLPATHS-LG v52488 (9) was used with default parameters to assemble the reads into scaffolds. The completeness of the assemblies was assessed using CEGMA (10). Protein-coding genes were predicted using MAKER (2.31.8) (11) in collaboration with AUGUSTUS (3.0.3) (12) and SNAP (2013-02-16) (13), both of which were trained with the Cryptococcus neoformans var. neoformans JEC21 sequence and GeneMark-ES (4.21) (14). The genes were functionally annotated using Sma3s (15) based on the UniProt-TrEMBL and UniProt-sprot (release 2015_11) databases. tRNAs, small noncoding RNAs, and transposons were also annotated using tRNAscan-SE (1.23) (16), Infernal cmscan (1.1.1) (17), RepeatMasker (open-4.0.5) (http://www.repeatmasker.org/), and RepeatRunner (18). The assemblies and annotations are summarized in Table 1.

**TABLE 1:** Assembly and annotation summary for genomic data of *D. crocea* and *D. aurantiaca*

Species	Strain	No. of reads	No. of scaffolds/contigs	Total length (Mbp)	Coverage (×)	Scaffold *N*_50_ value (kb)	GC content (%)	CEGMA score (%)	No. of protein-coding genes	No. of tRNAs	No. of small noncoding RNAs	No. of transposons
*D. crocea*	JCM 2961	35,136,662	26/86	20.6	176	1,954	53.2	95.6	8,753	35	135	126
*D. aurantiaca*	JCM 2956	24,122,574	52/139	19.3	112	1,282	53.6	94.8	8,106	37	207	206

Our data should help elucidate the properties of not only *Dioszegia* species but also other epiphytic phylloplane yeasts. Recently, Agler et al. (19) reported that *Dioszegia* is one of the microbial “hub” taxa that play important roles in the plant-microorganism relationship; thus, we believe that our genomic information will also have implications for the plant microbiome.

### Data availability.

The genome sequences have been deposited at DDBJ/EMBL/GenBank under BioProject no. PRJDB3718 with accession no. BCKK00000000 (for *D. crocea* JCM 2961) and PRJDB3721 with accession no. BCKN00000000 (for *D. aurantiaca* JCM 2956). The annotations are available via the JCM website (https://www.jcm.riken.jp/cgi-bin/nbrp/nbrp_list.cgi).
